# Influence of the epitaxial composition on N-face GaN KOH etch kinetics determined by ICP-OES

**DOI:** 10.3762/bjnano.11.4

**Published:** 2020-01-03

**Authors:** Markus Tautz, Maren T Kuchenbrod, Joachim Hertkorn, Robert Weinberger, Martin Welzel, Arno Pfitzner, David Díaz Díaz

**Affiliations:** 1 OSRAM Opto Semiconductors GmbH, Leibnizstr. 4, 93055 Regensburg, Germany; 2 Universität Regensburg, Universitätsstr. 31, 93053 Regensburg, Germany; 3 Friedrich-Schiller-Universität Jena, Humboldtstr. 10, 07743 Jena, Germany; 4 Departamento de Química Orgánica, Universidad de La Laguna, Astrofísico Francisco Sánchez s/n, 38206 La Laguna, Tenerife, Spain; 5 Instituto Universitario de Bio-Orgánica Antonio González, Universidad de La Laguna, Astrofísico Francisco Sánchez s/n, 38206 La Laguna, Tenerife, Spain

**Keywords:** etching, GaN, ICP-OES, KOH, LED

## Abstract

Roughening by anisotropic etching of N-face gallium nitride is the key aspect in today’s production of blue and white light emitting diodes (LEDs). Both surface area and number of surface angles are increased, facilitating light outcoupling from the LED chip. The structure of a GaN layer stack grown by metal organic chemical vapour deposition (MOCVD) was varied in the unintentionally doped u-GaN bulk region. Different sequences of 2D and 3D grown layers led to a variation in dislocation density, which was monitored by photoluminescence microscopy (PLM) and X-ray diffraction (XRD). Thin-film processing including laser lift off (LLO) was applied. The influence of epitaxial changes on the N-face etch kinetics was determined in aqueous KOH solution at elevated temperature. Inductively-coupled plasma optical emission spectroscopy (ICP-OES) was used to measure the etch progress in small time increments with high precision. Thereby, the disadvantages of other techniques such as determination of weight loss or height difference were overcome, achieving high accuracy and reproducibility.

## Introduction

The light emitting diode (LED) has become the basis of modern energy-efficient lighting technology over the last 30 years [1]. Especially the binary III–V semiconductor gallium nitride (GaN) is very useful for consumer lighting application. The high band gap energy of 3.4 eV at room temperature permits the production of blue and phosphor-converted white LEDs [2]. To reach a high internal quantum efficiency (IQE), crystal quality must be high in terms of low dislocation density [3]. Dislocations cause non-radiative recombination of induced electron hole pairs and thus lower efficiency. That is why considerable effort is put into the optimization of epitaxial growth to achieve the lowest possible dislocation density. Most often, GaN is produced in the LED industry by metal organic chemical vapour deposition (MOCVD) in a heteroepitaxial process on sapphire substrates [4]. The lattice parameter mismatch of ca. 13.8% between GaN and Al_2_O_3_ leads to a high dislocation density, if no strategy for defect reduction is applied. This spans the entire epitaxial layer. The most common strategy to bridge the structural mismatch is the application of AlN and GaN nucleation layers in between the sapphire substrate and bulk GaN [5–6]. Nucleation layers have the second but very important effect of controlling crystal orientation, which is commonly Ga-polar (0001) [7]. Other than nucleation layers, there are several more strategies for defect reduction. These all require the insertion of additional layers or layer transitions into the epitaxy stack. Thin-film technology is a common approach in industry to improve device performance. Thereby, a substrate with higher thermal conductivity, e.g., silicon or nickel, is bonded to the top p-contact of the LED structure [8]. The sapphire substrate is removed by laser lift off (LLO). This allows for higher operating power, which would otherwise lead to device overheating and degradation. After LLO, the N-polar GaN surface previously acting as interface between the sapphire and GaN epitaxy is uncovered on the surface.

In addition to IQE the extraction efficiency has to be considered. It represents the percentage of generated photons that can leave the LED chip and take part in light emission [9]. During extraction efficiency optimization, surface structuring of the N-polar GaN surface was found to increase light emission 4-fold, compared to the untreated surface [10]. Because of this, surface roughening is an integral part of flip-chip processing. The two approaches towards surface roughening are wet- and dry-chemical etching [11–12]. During wet etching, aqueous KOH and other alkaline or acidic solutions are commonly used to remove GaN from the N-polar surface in an anisotropic manner [13–14]. During KOH treatment hexagonal pyramids develop, based on the wurtzite type crystal lattice, thus increasing both the surface area and number of surface angles. The latter are beneficial to reduce the total internal reflection of photons emitted outside the small light escape cone of 23.6° between GaN and air [13].

In an earlier article, we reviewed the broad range of both material- and process-dependent influences on the etch rate [15]. In particular, growth conditions during epitaxy have been reported to have a strong impact on the etch rate. Material imperfections lower the resistance to corrosion of this otherwise relatively stable material. They also change the local electronic structure of the material. However, no precise understanding of the fundamental chemical reaction and the impact of multiple parameters has yet been generated. Many of the industrial processes still lack reproducibility and controllability, leading to a great potential for future cost-savings. Furthermore, researchers and high-tech companies in the LED market are currently developing the pixelated chips necessary for matrix lighting applications, e.g., in automotive front light applications [16]. A very important characteristic of such devices is pixel contrast. The latter is highly affected by the thickness of the unintentionally doped bulk GaN layer (u-GaN), which separates the AlN nucleation layer from the multiple quantum well (MQW) and is needed for dislocation density reduction. By means of etching, this layer can be partially removed and roughened later in chip processing. This leads to better device performance. However, to reach the precise etch depth and roughness, process control is of utmost importance. If too much GaN material is removed, valleys in between pyramids can cause shorts in the active layer. These either cause device failure immediately or reduce product lifetime. With that in mind, a precise analysis of etch behaviour is sought after, along with the knowledge of how the etch rate is influenced by different conditions during epitaxial growth.

In this report, we describe two major approaches to achieve better process control during wet-chemical GaN etching. Firstly, using inductively coupled plasma optical emission spectroscopy (ICP-OES) analysis, we present a powerful tool to monitor the average material removal with nm scale precision. In the second part, several different epitaxial layer stacks were designed and produced. These feature varying thicknesses and sequences of 2D and 3D grown u-GaN layers, with different dislocation densities. We applied ICP-OES to evaluate the effect of epitaxial variation on the etch rate in 30 wt % KOH solution. This facilitated an important tool to understand and control the etch rate for the development of new LED devices with high requirements concerning for stable wet-chemical surface structuring.

## Experimental

### Materials

KOH was purchased from BASF in Selectipur grade. Carl Roth supplied 36 wt % p.a. grade HCl and 69 wt % p.a. grade HNO_3_. NH_4_F was purchased from Honeywell Specialty Chemicals in Puranal grade. Planar Adamant Namiki sapphire wafers were used as growth substrates during epitaxy. Silicon wafers were purchased from Siltronic.

### Preparation of GaN layers

**Figure 1:**
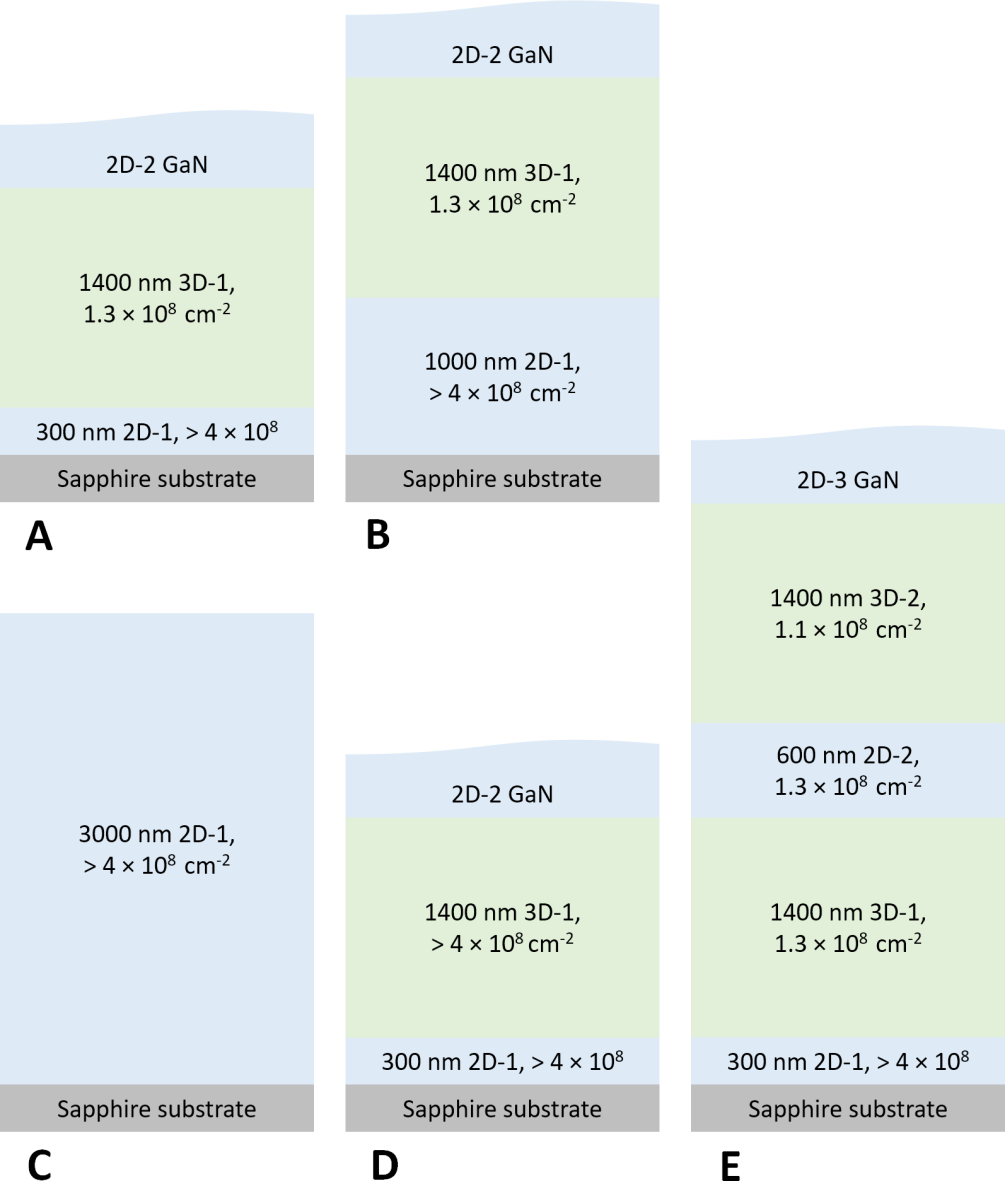
Epitaxial stack designs **A**–**E** grown by MOCVD. Different sequences of 2D and 3D GaN were prepared. Thereby, varying dislocation densities were achieved, which are indicated in the figure for each individual GaN layer. The dislocation density values were determined by PL and XRD.

Standard c-plane oriented GaN epilayers were grown on commercially available Veeco K465i and K700 tools. Epitaxy stacks **A**–**E** were prepared with different layer stacks (Figure 1). **A** acts as a reference sample in the following discussion. Usually 3D growth was applied in the layer closest to the substrate. We chose 2D growth to reach a high initial dislocation density and gain maximum variation in dislocation density. The bottommost 2D GaN layer was increased in thickness from 300 nm (**A**) to 1000 nm (**B**). A change to 3D growth conditions was applied to reduce defects caused by bending of dislocations into lateral direction. It is well known that 3D growth can be initiated by various growth methods, e.g., high pressure [17]. Sample **C** consists of a 3000 nm-thick single 2D GaN layer. **D** featured a modified 2D–3D transition leading to a higher number of dislocations penetrating the 2D–3D interface. In **E**, a sequence of two subsequent 2D–3D transitions was prepared, which were separated by a 3D and a 2D GaN layer with a combined thickness of 2000 nm. **A**, **C** and **E** contained a rudimentary MQW to allow for photoluminescence microscopy (PLM) analysis of dislocation density.

### Wafer processing

After growth with Ga-polarity, the Ga-face of epilayers were bonded to silicon carriers with a non-ohmic metal contact, to achieve electrical isolation and therefore no substrate influence on the etch process. The sapphire was removed by LLO to bear the N-polar 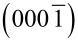 crystal facet. For maximum reproducibility, the epi-wafers were separated into 1 × 2 cm pieces. To protect the silicon substrate from etching in the hot KOH solution, a thin Pt layer was deposited on the reverse side. Prior to use, LLO residues were removed by treatment with a diluted aqueous NH_4_F solution for 10 min at room temperature and rinsed in deionized water (DIW).

### Etching

Etch experiments were performed in 50 mL polypropylene centrifuge vials under magnetic stirring. The temperature was precisely maintained with an uncertainty of ±1.5 °C, using an ethylene glycol heating bath and an IR laser thermometer. The credibility of temperature measurement was evaluated by comparison between IR and capillary thermometers. Before immersion of samples, the KOH solution was preheated for 10 min to find the balance between sufficient preheating and minimum water evaporation. In the case of etching at RT, we waited for 1 day to ensure temperature stabilization after mixing water and KOH. Etching was conducted comparing two sample types at once (Table 1). Etch time *t* was adapted to varying etch rates at different solution temperatures *T*. After Δ*t*, 250 µL of the KOH solution in which one sample chip was immersed was withdrawn and added to a mixture of 9.25 mL DIW and 500 µL 36 wt % HCl, for subsequent measurement. This dilution enabled ICP-OES analysis.

**Table 1:** Etch experiments conducted to determine GaN etch reaction kinetics. GaN chips were immersed in 30 wt % KOH solution for a total time *t*. After Δ*t*, samples were drawn for ICP-OES analysis.

Etch experiment	Samples	*T*/°C	*t*/min	Δ*t*/min

1	**A**, **B**	70	5	0.5
2	**A**, **B**	80	5	0.5
3	**B**, **C**	80	5	0.5
4	**A**, **C**	RT	50	5
5	**A**, **D**	80	5	0.5
6	**A**, **E**	80	50	5
7	**E**	70, RT	3 (70 °C) + 300 (RT)	–

### Methods

PLM images were recorded directly after epitaxy on an Olympus BX51 microscope combined with an Olympus U-RFL-T UV light source. For excitation, 408 nm wavelength light was used. X-ray diffraction (XRD) was measured on a Bruker/Jordan Valley QC Velox. Scanning electron microscopy (SEM) images were recorded on a Zeiss Gemini Leo 1530 microscope with an acceleration voltage of 2 keV and SE2 detection. For ICP-OES determination of the etch rate, a Thermo Fisher iCAP 6500 device was used. Plasma conditions were 1150 W power, 1.5 L min^−1^ support gas stream, 0.5 L min^−1^ spray gas stream and 12 L min^−1^ cooling gas stream. The Ga concentration obtained was averaged from three separate subsequent measurements of the identical dilution. The maximum integration time was set to 15 s. Both low and high standard solutions were prepared with identical concentrations of pure KOH and HCl to adapt the standard matrix to the samples and reach maximum precision. The Ga concentration in the high standard solution was varied between 2 mg L^−1^ and 5 mg L^−1^, depending on the expected Ga concentration of the sample. The diluted KOH sample was then measured to determine the Ga concentration based on the 417 nm emission wavelength of Ga. From the known bulk GaN density of 6.10 g cm^−3^ published in the literature, the respective average film thickness dissolved by etching was calculated [18]. Error bars were determined as mean ± standard deviation of the mean (SDM) for 3 GaN chips, etched under identical conditions. After 10 measurements, the ICP-OES tool was calibrated to circumvent tool drifting. To test the reproducibility of the method, four nominally identical GaN samples **A** were etched at RT for 1 h. After etching, three samples of each of the four etch solutions were withdrawn to reach a total number of 12 ICP-OES samples. Each of these samples was subsequently analysed four times and the average experimental GaN etch depth compared. Measurement precision was investigated by dissolving a known amount of elemental Ga in aqua regia for 3 d at RT. The resulting solution was diluted and KOH and HCl were added to adapt the matrices of standard and sample. The experimental Ga concentration was compared with the calculated concentration based on initial Ga weight. Titration for detection of changes in KOH concentration was conducted on a Metrohm Titrando system.

## Results and Discussion

### ICP-OES analysis of etch kinetics

**Figure 2:**
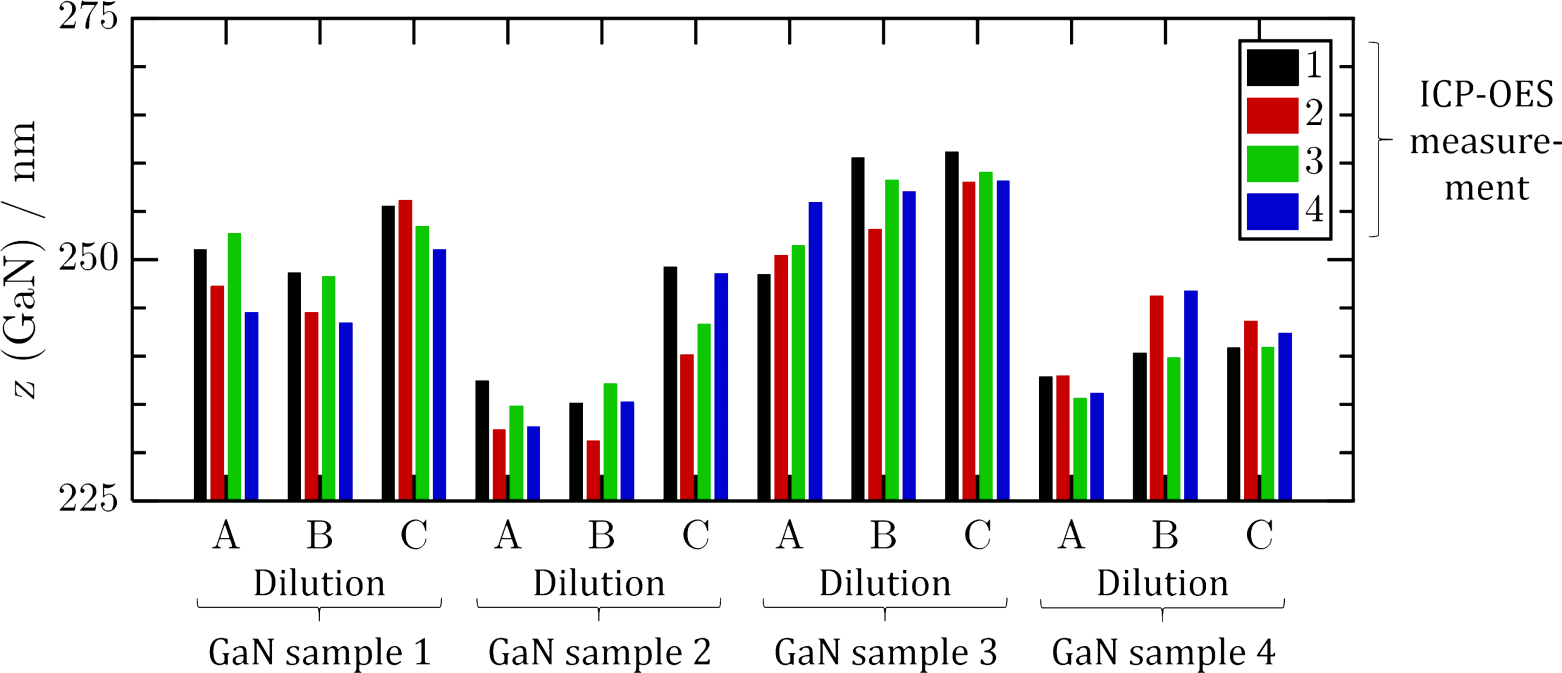
Reproducibility analysis of the ICP-OES method for determining etch depth *z* (GaN). Four GaN samples 1–4 were etched under identical conditions, i.e., in 30 wt % KOH at RT for 1 h. Each KOH etching solution was diluted three times to yield three sample solutions A, B and C. Each of the combined 12 sample solutions was measured four times.

Fourfold ICP-OES analysis of an identical KOH dilution stemming from a single GaN chip etched at RT for 1 h yielded 249 ± 3 nm average GaN removal and thus a relative error of 1% (mean ± SDM, *n* = 4; Figure 2). The measurement of different dilutions from the same KOH solution gave an etch depth of 250 ± 3 nm also leading to a relative error of 1% (mean ± SDM, *n* = 3). Finally, the analysis of four different GaN sample chips stemming from adjacent positions on the same wafer gave 246 ± 8 nm etch depth and a relative error of 3% (mean ± SDM, *n* = 4). On investigating ICP-OES reproducibility, the analysis error was lower than the variation in material. Maximum reproducibility in these terms was attained using sample pieces cut from a single wafer, taken from adjacent positions. The ICP-OES method thus had sufficient reproducibility to monitor the etch progress.

The stability of the KOH concentration during preheating was also monitored. Directly after preparation, titration with HCl gave a KOH concentration of 30.1 wt %. After 20 min heating to a temperature of 82 °C in a closed sample vial, the concentration had increased to 30.9 wt %. The reason was water evaporation and condensation on the vial sidewalls. As a GaN sample was etched in solution, after 10 min preheating and 10 min etching at 82 °C, the KOH concentration was determined as 30.93 wt %. Processing with open vials led to 31.7 wt %, 32.6 wt % and 33.4 wt % KOH after 15 min, 30 min and 45 min, respectively. This must be considered in the subsequent etch experiments. The increased KOH concentration during processing in aqueous solution at 80 °C might have lowered the etch rate. However, the temperature has an exponential influence on the GaN etch rate compared to the etch rate peak at ≈25 wt % KOH and the roll off towards higher concentrations [14]. Finally, 10 min preheating was maintained as the standard in the method to ensure stable temperature and thus a reproducible etching progress.

Measurement precision was determined by dissolving elemental Ga in aqua regia. Beforehand, dissolving a weighed amount of bulk u-GaN in 30 wt % KOH solution was not successful. Moreover, immersion of elemental Ga in 30 wt % KOH solution led to surface passivation after a short etch period. From the Ga mass dissolved in aqua regia, a concentration of 0.909 mg L^−1^ Ga after dilution was calculated. An ICP-OES measurement of this solution yielded a concentration of 0.902 mg L^−1^ and thus a mismatch of 0.8%. This showed that the adaption of the matrix between sample and standard solution was very precise.

With that method, two major disadvantages of previously reported approaches to monitor the etch progress were overcome. Compared to weight loss determination, our ICP-OES technique allowed for continuous monitoring. The process did not have to be interrupted, which would have immediately caused surface oxidation and measurement errors. Also, our method was superior to height profiling. In general, at a process temperature of 80 °C in half-concentrated KOH solution, etch rates of 50–400 nm min^−1^ are feasible [19]. After several minutes of etching, pyramidal features up to 2 µm in height may be formed, which can lead to a ≈400% uncertainty in etch depth. In our case, samples were drawn without the need to disrupt the etch process.

### Variation in epitaxial stack

The dislocation density of epitaxial layers **A**–**E** was monitored by XRD and PLM (Table 2). **A** acts as a reference sample in the reported experiments here. Crystal quality was improved slightly by increasing the 2D-1 thickness from 300 nm (**A**) to 1000 nm (**B**). This was indicated by a full width half maximum (FWHM) reduction of the (102) reflection from 170 to 161 arcsec. Therefore, it was assumed that the difference in overall layer thickness had no impact on its own. The insertion of a second 2D–3D interface (**E**) into the bulk u-GaN region yielded an even lower FWHM value of 142 arcsec. For **C**, no approach for dislocation density reduction was applied. A single 2D-1 layer with a thickness of 3000 nm was grown. This sample yielded a twice higher FWHM of 322 arcsec, the same result as for **D**. By modifying the growth conditions for **D** compared to **A**, the efficiency of dislocation bending in the lateral direction decreased to a point where there was no longer any difference between **C** and **D**.

**Table 2:** XRD FWHM of the (102) reflections determined from epitaxial layers **A**–**E** and PL dislocation densities determined from 5 sampling areas on PLM images by manual counting.

Sample	XRD (102) FWHM/arcsec	PLM dislocation density/cm^−2^

**A**	170	1.3 × 10^8^
**B**	161	–^a^
**C**	322	–^a^
**D**	324	>4 × 10^8^
**E**	142	1.1 × 10^8^

^a^Not determined separately due to similar XRD data as **A** and **D**.

**Figure 3:**
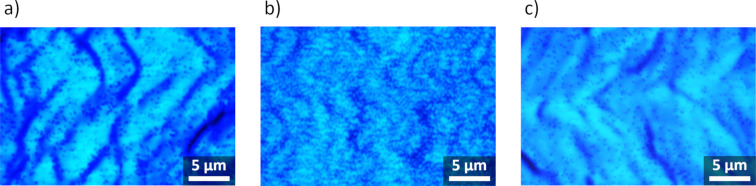
PL images of **A** (a), **D** (b) and **E** (c) used for determination of dislocation density.

Regarding PLM analysis, the number of dislocations reaching the uppermost epitaxial layers of **A** was determined as 1.3 × 10^8^ cm^−2^ (Figure 3). The decrease in FWHM found for **E** corresponds well to the lower PLM determined dislocation density of 1.1 × 10^8^ cm^−2^. Compared to this value, with no functional reduction in dislocations, 3.6 × 10^8^ cm^−2^ was determined for **C** and **D**. However, this did not include the clustering of dislocations in PLM emission and thus represents a minimum value. The actual dislocation density was assumed to be in a range between 4 × 10^8^ cm^−2^ and 1 × 10^9^ cm^−2^ and is thus expressed as >4× 10^8^ cm^−2^ in Figure 1 and the following discussion. XRD refers to overall thickness, whereas PLM revealed the number of dislocations in the uppermost bulk GaN region that spanned the entire epitaxial layer up to the MQW region. As the respective layers **B** and **C** showed similar XRD FWHM results as **A** and **D**, the dislocation density values given in Figure 1 were transferred.

### Effect of epitaxial changes on etch kinetics

**Figure 4:**
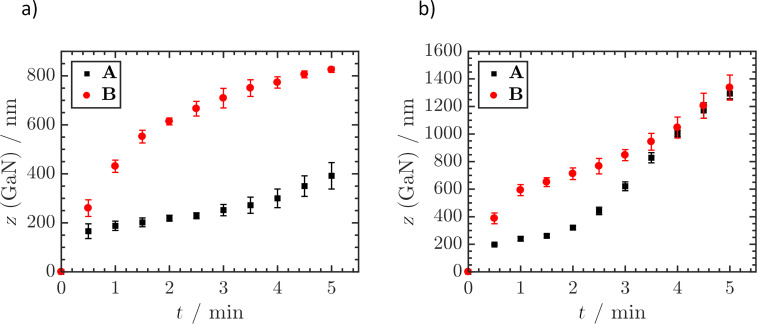
Average GaN removal *z* (GaN) of **A** and **B** during etching in 30 wt % KOH at 70 °C (a) and 80 °C (b) determined by ICP-OES. Values were determined as mean ± SDM, *n* = 3.

The etch kinetics of **A** and **B** were compared at 70 °C and 80 °C (Figure 4). The fastest etching was observed at the beginning of each experiment. The etch rates hereby were comparable, which is feasible because the first 300 nm of material were identical in terms of epitaxy design. At both temperatures tested, **A** became saturated at 240 ± 17 nm to form a plateau region. Considering that LLO removes up to 80 nm of material, this plateau formed at the interface between 2D-1 and 3D-1. Depending on solution temperature, this plateau could be approached with high precision. Especially at 70 °C, no further material removal was observed over a time span of 1.5 min (Figure 4a). The reproducibility of etching was very high, as indicated by the small error bars. At 80 °C, both samples were etched faster (Figure 4b).

**Figure 5:**
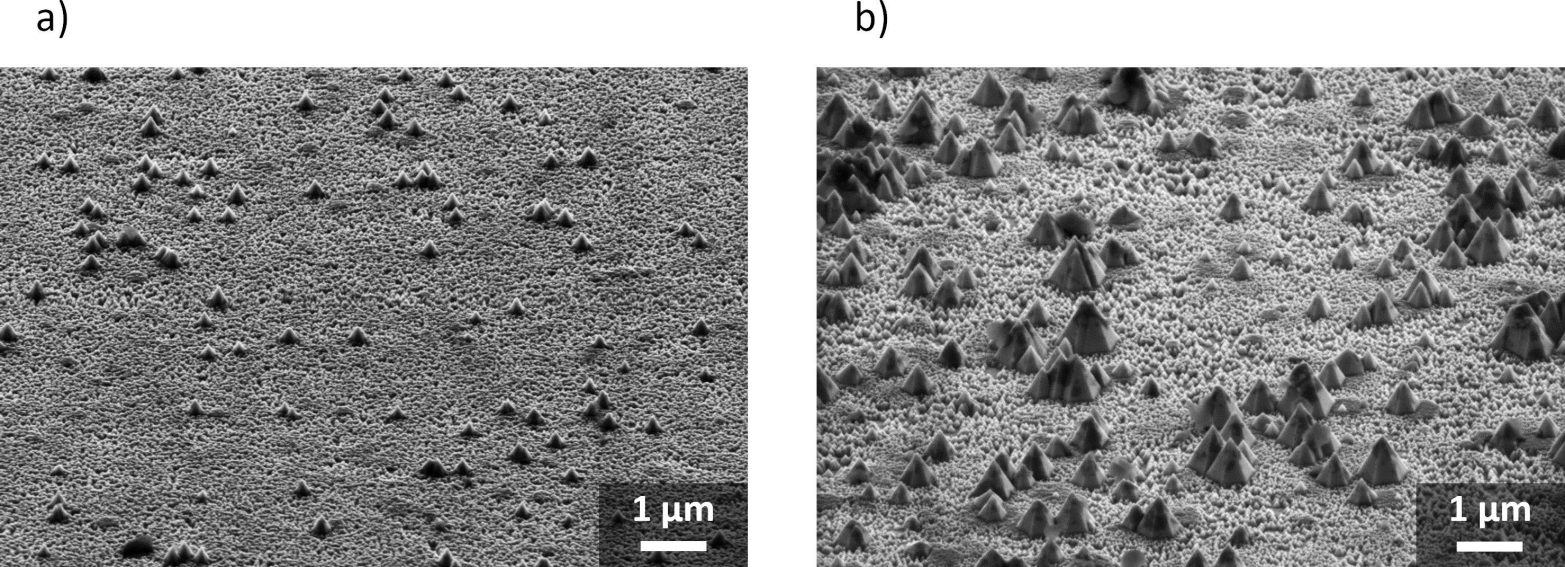
SEM images of **A** after 60 min etching in 30 wt % KOH solution at RT (a), and of **B** after 2 min etching in 30 wt % KOH solution at 80 °C (b). Images were recorded at an angle of 60°.

In the etch kinetics of **B** no distinct plateau formation was observed at the 2D–3D transition. However, SEM analysis provided evidence of the formation of a flat plane due to a temporary etch stop at the 2D–3D transition, in both samples (Figure 5). The reason for the missing plateau in the etch kinetics of **B** is that the average pyramid size increased to several hundred nm at this point. Therefore, the pyramids spanned multiple layers at once. In some regions the transition was already completed, leading to further etch progress. In contrast, etching in other sections of the sample was still slowed down by the transition. In other words, the plateau was smeared out due to the formation of large pyramids in case of **B**. After 4 min etching at 80 °C the etch depth of both **A** and **B** coalesced, yielding values of 1294 ± 38 nm and 1337 ± 91 nm after 5 min etch time, respectively.

**Figure 6:**
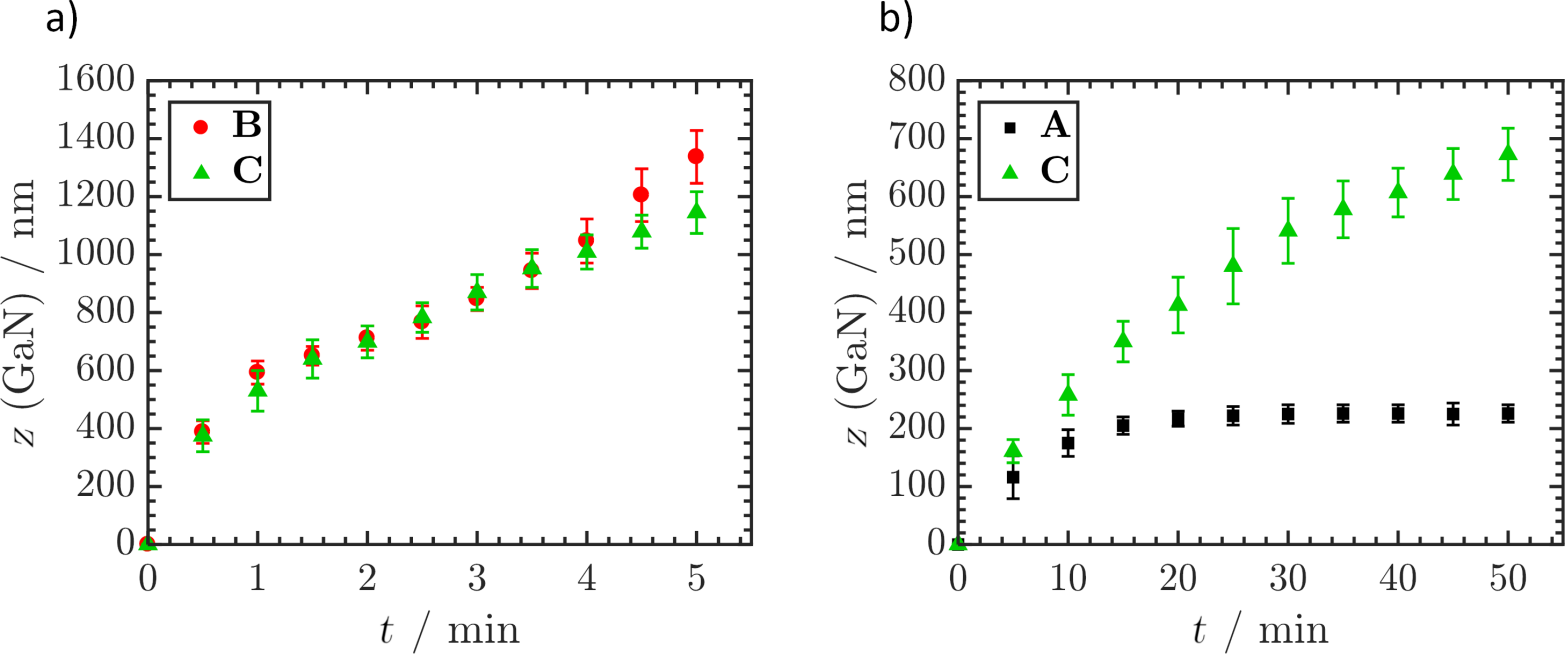
ICP-OES determined average GaN removal z (GaN) of **B** and **C** during etching in 30 wt % KOH at 80 °C (a) and **A** and **C** during etching in 30 wt % KOH at RT (b). Values were determined as mean ± SDM, *n* = 3.

The etch progress of **C**, which was intentionally designed without a 2D–3D transition, was identical to **B** up to an etch depth of 952 ± 65 nm (Figure 6a). From this point on the etch rate was lower, showing that 2D GaN in general is etched at a slower rate than 3D GaN. The transition of 2D to 3D in all samples caused an increased etch rate once the plateau region was crossed. Etching of **A** and **C** at RT revealed that the 2D–3D interface present in **A** could not be overcome. This was previously indicated by the pronounced plateau formation observed in **A** at 70 °C. Compared to the etch saturation of **A**, the missing interface in **C** led to a continual etch progress up to 50 min processing and a material removal of 728 ± 54 nm. After that, the process was concluded. By suitable process control, etching can be terminated at the 2D–3D interface. This therefore constitutes a useful tool for the selective dissolution of distinct epilayers exposed on the wafer surface. The finding that the interface leads to plateau formation or even etch stop up to this point was not expected. The evidence that 3D GaN is actually etched faster than 2D GaN pointed to a simple etch rate increase rather than an etch stop.

**Figure 7:**
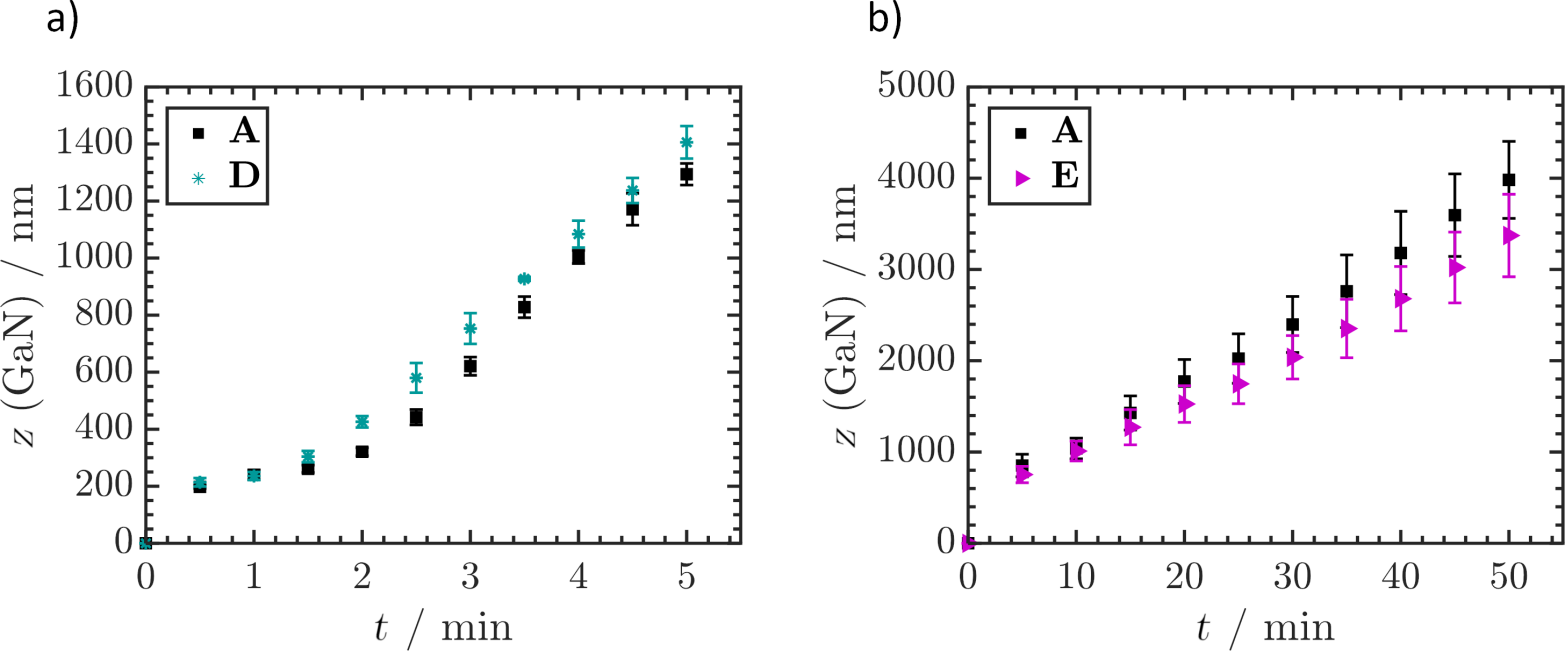
ICP-OES determined average GaN removal *z* (GaN) of **A** and **D** (a) and **A** and **E** (b) during etching in 30 wt % KOH at 80 °C. Values were determined as mean ± SDM, *n* = 3.

To determine the mechanism behind the etch stop or plateau formation occurring at the 2D–3D interface, **D** was designed. A reduction in dislocation density was circumvented while maintaining the 2D–3D transition. Etching of **D** behaved similarly to **A,** proving that the acquired difference in dislocation density did not influence the etch rate (Figure 7a). This behaviour is remarkable as it seemingly contradicts the general understanding that highly defective layers are etched faster than perfect single crystalline material [20]. It was also observed that the transition plateau region was passed in a shorter time. A constant etch rate was reached after 1.5 min etching, compared to 2 min in the case of **A**.

**Figure 8:**
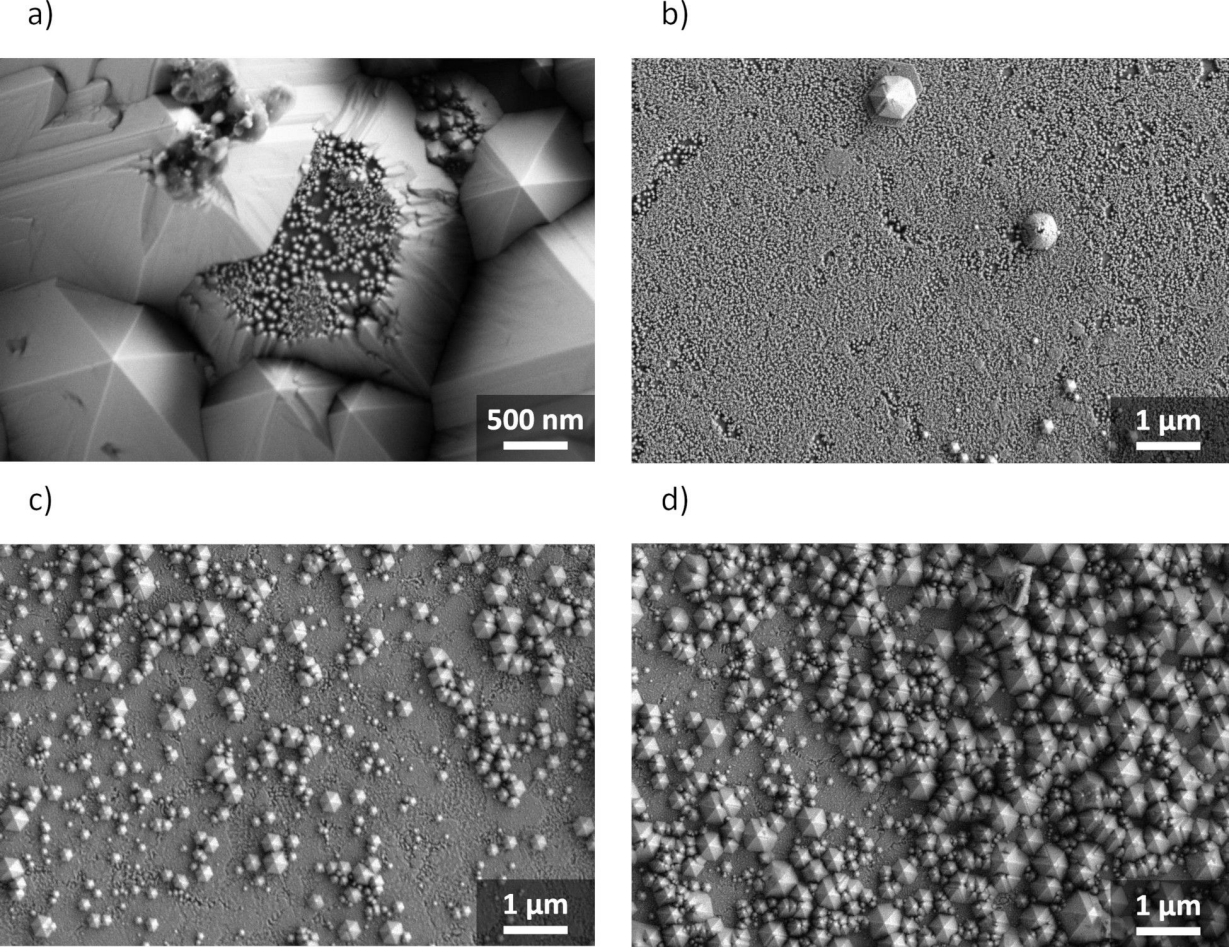
SEM image of the second 2D–3D transition plateau on pyramid top of **E** after etching in 30 wt % KOH solution at 80 °C for 50 min (a). SEM images of different sections of identical sample **E**, after etching in 30 wt % KOH solution at 70 °C for 3 min and subsequently 5 h at RT (b–d).

To provide further evidence, **E** was included in the study comprising two transitions from 2D to 3D GaN. An etch time of 50 min was necessary to remove sufficient material to approach the second 2D–3D transition. After the long process time in several sections of the samples, the entire epitaxial layer was removed. This exposed the underlying metal layer. Local inhomogeneity in etching increased the error compared to the previous experiments. The impact of local inhomogeneity was greater for longer process times, since initially minor etch rate variations caused a significantly greater difference in material removal after 50 min. The first 2D–3D transition occurred within the first minutes of etching, so it is not visible in the diagram. The second 2D–3D transition was at a theoretical depth of 2300 nm into the material, minus 80 nm of maximum LLO removal. As stated earlier, the pyramid sizes at this point spanned several epitaxial layers, blurring any distinct plateau formation. However, SEM of samples etched for 50 min revealed that the second 2D–3D transition also led to a temporary etch stop layer, despite the dislocation density only decreasing from 1.4 × 10^8^ cm^−2^ to 1.1 × 10^8^ cm^−2^. Even though selectivity was low due to the wide *z*-amplitude of pyramidal morphology, some pyramids showed the etch plateau instead of sharp tips (Figure 8a). To resolve the etch stop layer, **E** was etched at 70 °C for 3 min to overcome the first 2D–3D transition. Subsequently the sample was treated with 30 wt % KOH at RT for 5 h, which led to a combined material removal of 1161 ± 127 nm (mean ± SDM, *n* = 3) determined by ICP-OES. SEM analysis afterwards showed free standing GaN pyramids in several sections of the sample surface (Figure 8b). Other regions of the material were less fully etched due to local inhomogeneity, thus reducing the average material removal determined by ICP-OES (Figure 8c,d). The exact reason for plateau formation at the interface between 2D and 3D GaN layers could not be determined during the course of this study and will be further investigated in another key part of our research.

## Conclusion

This work demonstrated two powerful tools to enhance the controllability of wet chemical roughening of N-face GaN in aqueous KOH solution. First, an ICP-OES method was developed to monitor the average etch depth during the process. This monitoring has a major advantage compared to weight loss determination, since it can be conducted steadily without the need to interrupt the etch process due to rinsing and drying the samples. Consequently, outstanding precision with a relative error of 1% was achieved, in comparison with determination of weight loss or height difference.

Secondly, greater understanding was reached of how different conditions during bulk GaN growth influence the etch rate. The bottommost 2D-1 GaN layer of epitaxial samples **A**–**E** had a dislocation density >4 × 10^8^ cm^−2^. Etch saturation was observed at the first 2D–3D layer transition that occurred after 300 nm (**A**). Indeed, SEM also showed the formation of an etch stop layer. At this interface, the dislocation density was reduced to 1.3 × 10^8^ cm^−2^. Penetration was highly dependent on solution temperature. At RT, no crossing was observed, while at 70 °C and 80 °C it took 2.0 min and 1.5 min, respectively. The subsequent 3D GaN showed a constant etch rate. When the thickness of 2D-1 was increased to 1000 nm (**B**), no plateau was seen in the etch kinetics at the 2D–3D transition due to the large pyramid sizes spanning multiple epitaxial layers at once. However, SEM showed a flat etch stop layer. **D** and **E** contained 2D–3D transitions without significant dislocation reduction and also showed plateau formation. Therefore, the transition itself caused the etching to stop rather than the difference in dislocation density. **C** did not contain a 2D–3D transition and exhibited almost identical etch behaviour to **B,** rather than a constant etch rate. Linear etching was only achieved when the equilibrium between 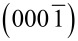 and 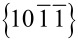 crystal facets was reached.

Applying temperature dependent crossing of 2D–3D transitions, the second 2D–3D transition of **E** became selectively exposed by first etching in 30 wt % KOH at 70 °C for 3 min. This led to penetration of the first 2D–3D interface. Subsequently, etching at RT for 5 h dissolved both 3D-1 and 2D-2 layers to eventually terminate at the second 2D–3D interface, which was successfully verified by SEM. This confirms the possibility of homogenous removal of material from the entire GaN surface by means of a previously unmatched process control.
